# Rabies surveillance in Madagascar from 2011 to 2021: can we reach the target?

**DOI:** 10.3389/fvets.2023.1270532

**Published:** 2023-10-12

**Authors:** Soa Fy Andriamandimby, Marie Hermelienne Volasoa, Nivohanitra Perle Razafindraibe, Dany Bakoly Ranoaritiana, Mino Harimbola Razafindramparany, Théophile Rafisandratantsoa, Lalaina Arivony Nomenjanahary, Naltiana Rakotondrabe, Mamitiana Aimé Andriamananjara, Hélène Guis, Vincent Lacoste, Anou Dreyfus

**Affiliations:** ^1^Unité de Virologie, Institut Pasteur de Madagascar, Antananarivo, Madagascar; ^2^Département des Enseignements des Sciences et de Médecine Vétérinaire, Université d’Antananarivo, Antananarivo, Madagascar; ^3^Direction des Services Vétérinaires, Service de Surveillance et de la Lutte contre les Maladies Animales, Antananarivo, Madagascar; ^4^Direction de la veille sanitaire, de la surveillance épidémiologique et de la riposte, Ministère de la santé publique, Antananarivo, Madagascar; ^5^CIRAD UMR ASTRE, Antananarivo, Madagascar; ^6^Université de Montpellier, CIRAD, INRAE, Montpellier, France; ^7^Unité d’Epidémiologie et de Recherche Clinique, Institut Pasteur de Madagascar, Antananarivo, Madagascar; ^8^Epidemiology and Public Health Unit, Institut Pasteur du Cambodge, Phnom Penh, Cambodia

**Keywords:** rabies, surveillance, Madagascar, direct fluorescent antigen test, dogs

## Abstract

Rabies is endemic in Madagascar and a neglected disease. The aim of this study was to summarize human and animal rabies surveillance activities in Madagascar from 2011 to 2021. Samples from terrestrial mammals and humans were tested for rabies virus infection using direct fluorescent antibody, RT-PCR and virus isolation by the National Reference Laboratory (NRL) for rabies at the Institut Pasteur de Madagascar. Among 964 animal and 47 human samples tested, 66.7 and 70.2% were positive, respectively. The NRL received these suspect rabies samples from 48 of 114 districts of Madagascar. Most of them were submitted from the district of the capital city Antananarivo (26.3%) and mainly from its region Analamanga (68.9%). Animal samples were mainly from dogs (83%), cats (9.5%) and cattle (5.8%). Pigs, lemurs, goats accounted for less than 1%. During the 11 years of surveillance, 48 human skin and/or brain biopsy samples were received from 20 districts, mainly from Antananarivo and its surroundings (*N* = 13), Toamasina and its surroundings (*N* = 8) and Moramanga (*N* = 6). The high positivity rate for all species and the non-homogeneous spatial distribution of samples suggests substantial underreporting of rabies cases. There is a clear need to better understand the reasons for underreporting and prioritize rabies surveillance, prevention and control in Madagascar, with improvements in budget, education and infrastructure. A joint animal and human health rabies control program including vaccination of at least 70% of the dog population, is needed to achieve the goal of eliminating dog-transmitted human rabies by 2030 from Madagascar.

## 1. Introduction

Animal and human rabies are preventable through vaccination and vaccine is available and safe (1). Nevertheless, the rabies virus remains endemic in many parts of the world and still represents an important threat to public health (1). For decades, over 99% of reported human cases worldwide are dog-transmitted. Despite the scientific literature reporting 59,000 annual deaths due to rabies, the perception of the importance of rabies control by policy makers, public health workers and even veterinarians may be different from country to country (2, 3). As a result, rabies mainly affects poor and vulnerable populations in rural areas due to ignorance and, in some cases, misinformation (1, 4). In this context, the World Health Organization (WHO), the World Organization for Animal Health (WOAH), the FAO and the Global Alliance for Rabies Control (GARC) adopted in 2015 a global initiative to eliminate human deaths from dog-mediated rabies by 2030 (5). Eliminating rabies in dogs is the optimal control method for preventing the spread of the disease (6–9). To reach this goal, accurate data on the incidence and true burden of rabies needs to be collected. It is therefore important to strengthen rabies surveillance and control at the local and national levels to provide robust estimates that will be used by policy makers (10).

In Madagascar, rabies remains a neglected disease. The first national vaccination campaign against rabies took place in 2019. It was organized by the Ministry of Agriculture and Animal Husbandry (MAAH), which had received 100,000 doses of animal vaccines from the Global Alliance for Rabies Control (GARC). However, the COVID-19 pandemic and other logistical issues hampered this momentum. As a result, no exact data on dog vaccination coverage is available to date. While very few dogs are vaccinated in Madagascar, post-exposure prophylaxis (PEP) for humans is in place. PEP is available in a network of 31 anti-rabies treatment centers (CTAR) distributed throughout the country. A CTAR is present in each district capital of each of the 22 administrative regions. A further nine CTARs are located in the most densely populated landlocked districts. All CTARs are supplied with rabies vaccine free of charge by the Institut Pasteur de Madagascar (IPM) in Antananarivo. It is the responsibility of the manager of each CTAR to obtain supplies from IPM, often at his/her own expense. As a result, while CTARs in major urban centers have large visitor numbers and provide PEP for free, patients in some remote rural areas can be asked to pay a financial contribution for PEP services in order to cover part of the transport costs (11). The fees charged are left to the discretion of each CTAR and no official information is available on their amount. Overall, in 2018 and 2019, about 15,000 patients per year required PEP nationwide, with 42% of patients visiting the major CTAR located at IPM in the capital city Antananarivo (11).

Rabies is a notifiable disease in Madagascar. Its surveillance is exclusively passive and involves three entities: the National Reference Laboratory (NRL) for rabies hosted by the Virology Unit at IPM, the MAAH and the Ministry of Public Health (MoPH). Animal and human rabies diagnosis is free of charge and financially supported by IPM. The MAAH manages the surveillance of animal diseases *via* the Madagascar Animal Disease Surveillance network while the MoPH is responsible for human disease surveillance. The NRL notifies both government bodies of all confirmed rabies cases. Upon receipt of a rabies notification by the NRL, the MoPH and MAAH work together to ensure that the bitten person receives PEP at one of the 31 CTARs. However, at all levels of the health system, any medical staff receiving patients who have been bitten or scratched should refer the patient to a CTAR to receive PEP even before the suspected animal is confirmed to have rabies, to be certain that they receive PEP during the incubation period. In theory, a dog suspected of having rabies or of having bitten a person is quarantined and remains under observation for 15 days by a veterinarian. If the animal develops rabies, the veterinarian euthanizes it and takes a sample for a confirmatory diagnosis at the NRL. However, animals are more often killed immediately or not handled at all.

This report summarizes rabies surveillance activities in Madagascar from 2011 to 2021. The aim is to provide an update of the rabies surveillance data since the publication of the previous report (2005–2010) (12), and to identify the specific factors associated with the poor performance of rabies surveillance.

## 2. Materials and methods

### 2.1. Diagnostic activities

Animal samples (entire head, brain samples or cadavers of terrestrial non-flying mammals) are received at the NRL at ambient temperature or ideally at +4°C. At the time of writing, there is no coordinated system for sending suspected rabies samples to the NRL. Samples are sent either by veterinarians or their assistants, animal health officers, or directly by animal owners or any person exposed at their own expense. In the case of group bites involving one or more stray dogs, a local administrative agent will submit the samples after catching the dog(s) (12). To limit the sending of large samples (brain sample vs. animal head) and reduce shipping costs, the NRL team has been organizing training courses on sampling techniques since 2019.Human samples (post-mortem skin biopsies, saliva, or brain biopsies taken from the nape of the neck) (13) are sent at ambient temperature or ideally at 4°C by the MoPH staff after the hospital team has notified a suspect case.

A sampling form has been issued by the NRL. In most cases, the laboratory technician receiving the sample fills in the information sheet based on the information provided by the remitter. The information is then recorded into a standardized database. The information collected includes the transmitter (veterinarian or other), the name and detailed address of the owner, if available, the animal’s rabies vaccination status and related information, disease history, symptoms reported to assess clinical suspicion of furious or paralytic rabies, the aggressiveness of the animal, whether or not it has bitten, and the circumstances of the bite.

Direct fluorescent antigen test (DFAT) is the reference technique used at the NRL. All biopsy brain samples are first tested by DFAT. For any negative test result, a second test is performed: either an isolation attempt in cell culture (Neuro-2A) (14), or RT-PCR (13, 15, 16). A second negative result by one of these two other tests is definitive. Human skin biopsies are tested by RT-PCR.

### 2.2. Statistical analyses

We performed a descriptive analysis of the data, calculating absolute numbers and proportions using R version 4.3.1. software. The association between descriptive category variables and diagnostic test results was calculated using the chi square test with 95% confidence intervals.

## 3. Results

From 2011 to 2021, the NRL received a total of 987 samples from animals suspected of rabies and 48 samples from suspected human cases, of which 964 (97.7%) and 47 (97.9%) were eligible for testing, respectively. The remaining samples were not suitable for testing due to inadequate transport conditions. The annual number of samples submitted to the NRL varied from 55 to 151 between 2011 and 2021. Animal samples were mainly from dogs (*N* = 819, 83%), cats (*N* = 94, 9.5%) and cattle (*N* = 58, 5.8%). Other species (pig (*n* = 3), lemurs (*n* = 2), goat (*n* = 1)) accounted for less than 1% over the study period (Table 1). Among animal samples, 863 (87.4%) were from animals with owners.

**Table 1:** Description of animal sample (*N* = 987) characteristics received at the National Reference Laboratory for rabies in Antananarivo, Madagascar from 2011 to 2021.

	Female	Male	Total
	*N* (%)	*N* (%)	*N* (%)
	342 (41.4)	485 (58.6)	987
*Animal species*			
Cat	39 (48.1)	42 (51.9)	94 (9.6%)
Cow	30 (57.7)	22 (42.3)	58 (5.9%)
Dog	269 (39.2)	418 (60.8)	819 (83.4%)
Goat	1 (100.0)	0 (0.0)	1 (0.1%)
Pig	2 (100.0)	0 (0.0)	3 (0.3%)
Lemur	0 (0.0)	0 (0.0)	2 (0.2%)
Rat	0 (0.0)	0 (0.0)	5 (0.5%)
*Age category*			
<1 year	112 (45.2)	136 (54.8)	356 (40.7%)
1–3 years	90 (38.8)	142 (61.2)	241 (27.6%)
3–6 years	80 (41.2)	114 (58.8)	199 (22.8%)
>6 years	32 (42.7)	43 (57.3)	78 (8.92%)
*Biting animal*			
No	79 (48.5)	84 (51.5)	184 (19.3%)
Yes	254 (39.5)	389 (60.5)	768 (80.7%)
*History of rabies vaccination*			
No	322 (42.5)	435 (57.5)	899 (93.9%)
Yes	14 (25.9)	40 (74.1)	58 (6.06%)
*Death circumstances*			
Euthanized	156 (41.5)	220 (58.5)	453 (53.4%)
Spontaneous	140 (41.3)	199 (58.7)	395 (46.6%)
*Owned animal*			
Yes	311 (41.0)	447 (59.0)	863 (87.4%)
No	31 (44.9)	38 (55.1)	124 (12.6%)
*Symptoms of rabies*			
No	95 (38.5)	152 (61.5)	269 (28.1%)
Yes	241 (42.4)	327 (57.6)	687 (71.9%)
*Submitter*			
Non-veterinarian	210 (39.4)	323 (60.6)	637 (64.5%)
Veterinarian	132 (44.9)	162 (55.1)	350 (35.5%)

Missing data: 160 (16%) on gender, 113 on age, 5 (0.5%) for animal species, 35 (3.5%) for biting information, 30 (3%) on anti-rabies vaccination, 31 (3.1%) on rabies symptoms and 139 (14.1%) on death circumstances.

### 3.1. Circumstances of sampling and sending samples to the NRL

Samples were taken after a bite or attack (768, 77.8%) or in the event of rabies symptoms (687, 69.6%) (*p*-value < 0.001). Only 350 (35.5%) samples were sent by a veterinarian or his/her collaborators. In most cases, it was the owners or the bitten victims who sent the samples to the NRL. The majority of samples sent by veterinarians (72%) were from livestock suspected of having rabies. When the samples arrived at the NRL, we had no information on the gender of the animal for 160 (16%) of them, on the species for 5 (0.5%), on the characteristics of the bite for 35 (3.5%), on the rabies vaccination status for 30 (3%) or on the circumstances of death for as many as 139 (14.1%) of them. When veterinarians submitted samples, more data on biting behavior was missing (19/350 (5.4%) vs. 16/637 (2.5%)) (*p* = 0.01). The proportion of animals showing rabies symptoms was higher in ownerless animals (84.3% vs. 70.2%, *p* = 0.002). Until 2020, most animal samples (70.5%) sent to the NRL were heads, whereas in 2021, only brain samples were sent to the NRL.

### 3.2. Geographical origin of samples

#### 3.2.1. Animal samples

**Figure 1:**
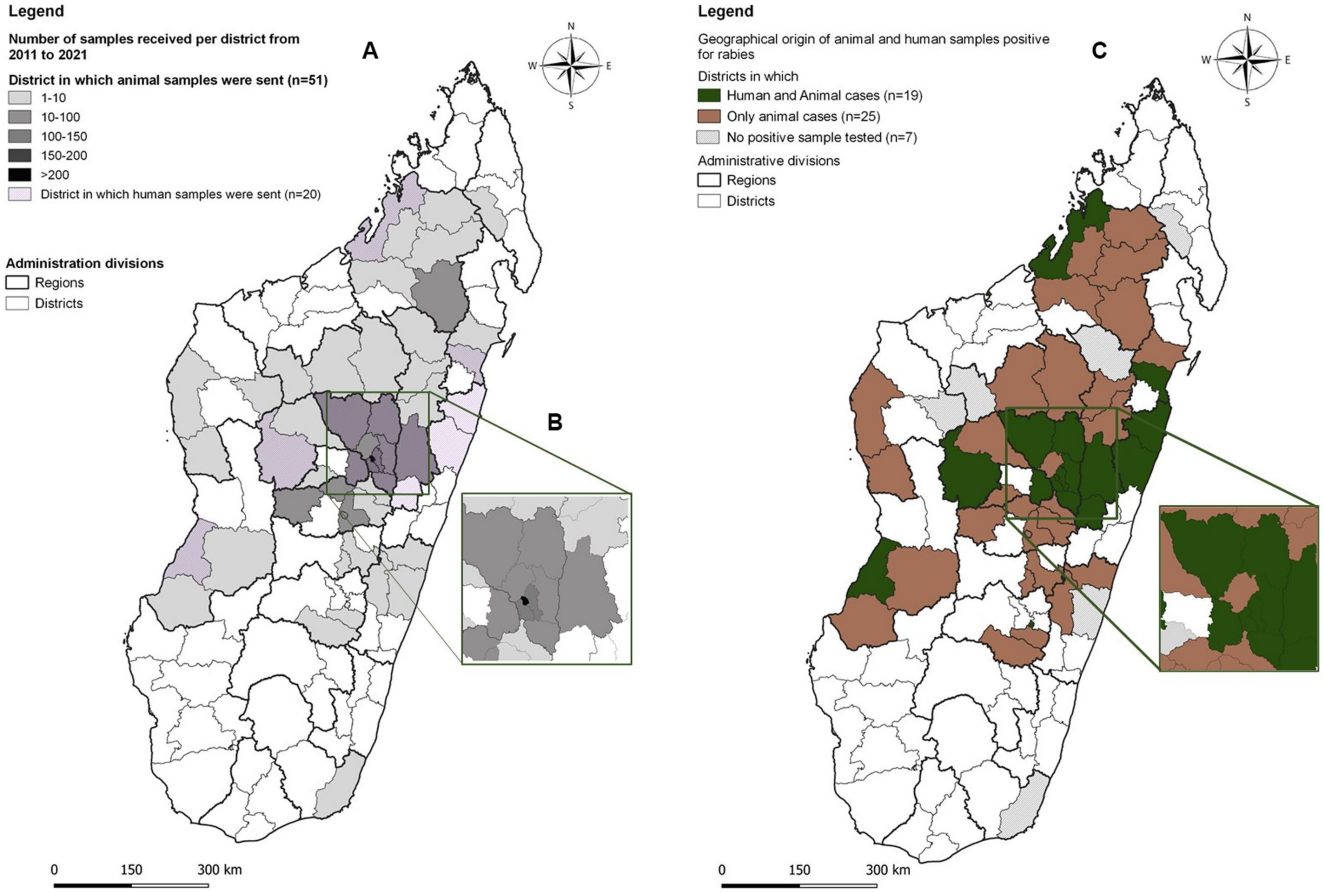
**(A)** Geographical origin and number of suspect rabies samples sent to the National Reference Laboratory for rabies, Antananarivo, Madagascar, received between 2011 and 2021. **(B)** Suspect samples for rabies received from the Analamanga region (Capital). **(C)** Origin of animal and human samples positive for rabies.

The NRL received suspect rabies samples from 51 of 114 districts of Madagascar. However, the majority of them were submitted from the district of the capital city Antananarivo (n = 259, 26.3%) and mainly from the region Analamanga, the region of the capital city (68.9%) (Figure 1). Rabies circulation was confirmed for 44 districts, where at least one received sample was confirmed to be rabies positive. For the remaining seven districts, the NRL only received one sample for each district, which tested negative.

#### 3.2.2. Human samples

During the 11 years of surveillance, 48 human skin and/or brain biopsy samples were received from 20 districts (Figure 1). As in the case of animals, the majority of samples came from the capital and its surroundings (*N* = 13), the city of Toamasina (East Coast) and its surroundings (*N* = 8), Sainte-Marie (*N* = 1) and Moramanga (*N* = 6).

### 3.3. Diagnostic results

#### 3.3.1. Animal samples

**Figure 2:**
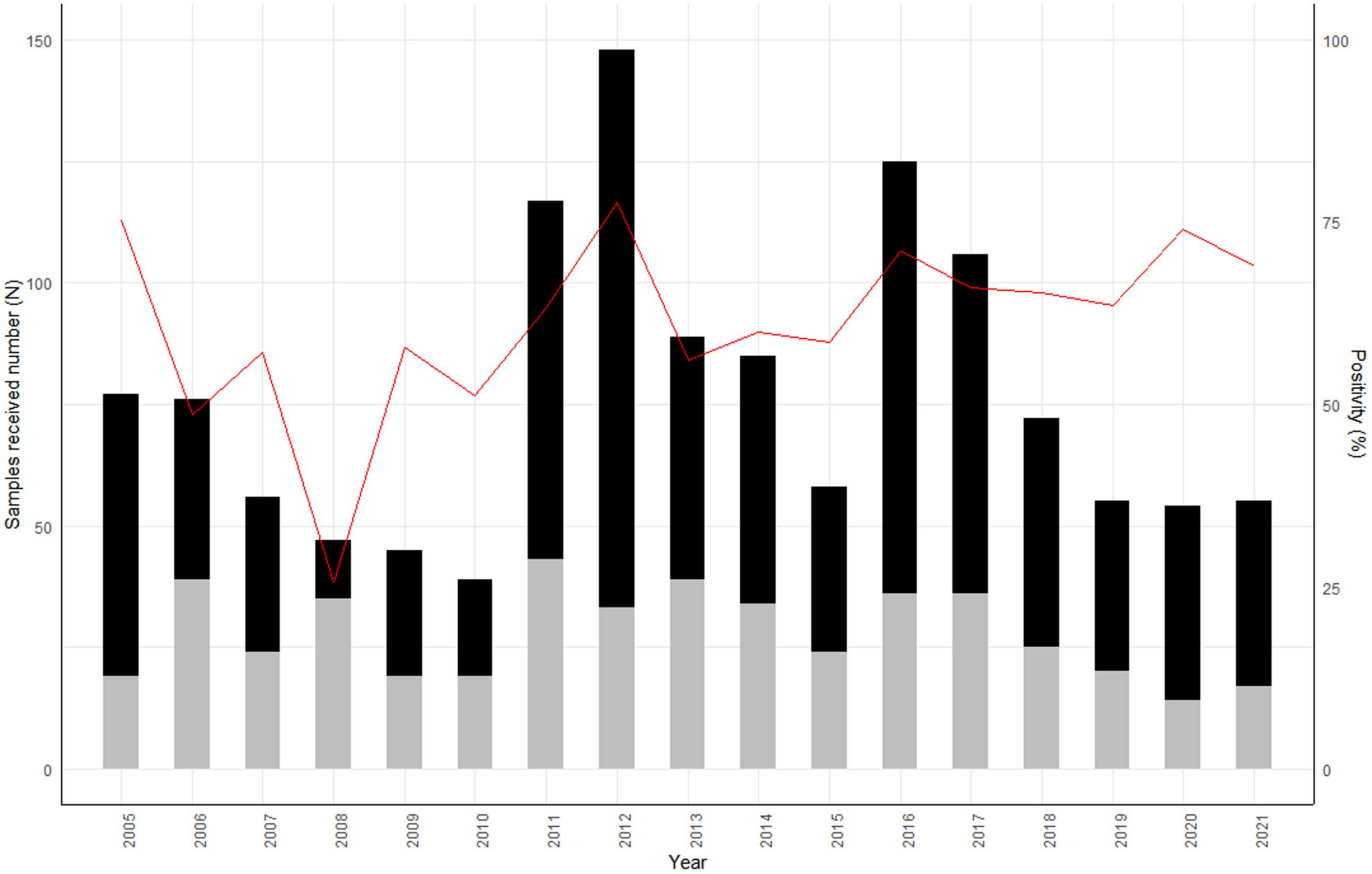
Number of animal samples received annually by the National Reference Laboratory for rabies from 2005 to 2021, Madagascar. The data from 2005–2010 were analyzed and published by Reynes et al. (12). Black: Positive samples, Grey: Negative samples, Red line: % of positivity.

Overall, of the 964 samples meeting the test criteria, 643 (66.7% (95% CI: 63.9–69.7) tested positive by DFAT, by cell culture (Neuro-2A) or by RT-PCR. The percentage of positivity ranged from 56.8% (95% CI: 45.8–67.2) in 2013 to 77.7% (95% CI: 69.9–89.9) in 2012 (Figure 2). Positivity was significantly higher in livestock (*p* < 0.001) (cattle (51/57, 89.5% (95% CI: 78.6–96.5), pigs (3/3, 100, 95% CI: 30.0–100), and goats (1/1, 100, 95% CI: 5.5–100), followed by dogs (564/798, 70.7, 95% CI: 67.4–73.0) and cats (22/93, 23.3, 95% CI: 15.7–33.8).

#### 3.3.2. Human cases

Of the 48 human samples received, 47 were tested and rabies infection was confirmed in 33 individuals (70.2, 95% CI: 55.1–82.7) in 19 districts (Figure 1).

### 3.4. Association of sample characteristics and a positive diagnostic rabies result

Table 2 summarizes the association between descriptive category variables and diagnostic test results. We observed a statistically significant association (*p* < 0.001) between a positive test result and a sample originating from a biting animal, or an animal showing clinical symptoms. The proportion of positive test results was significantly higher in livestock than in pets (*p* < 0.001). Only 6.2% of tested animals had a history of vaccination, and of those vaccinated 22/56 (37.9%) were positive versus 598/878 (68.1%) among animals with no history of vaccination (p < 0.001). A higher number of positive cases were observed among samples submitted by veterinarians than among those submitted by bite victims and/or their relatives (*p* < 0.001).

**Table 2:** Test results of rabies diagnostics^a^ on animal samples (*N* = 964) stratified by categorical variables^b^, collected from 2011 to 2021 at the National Reference Laboratory for rabies in Antananarivo, Madagascar.

		Positive	Negative	Total	*p*-value
		*N* = 643 (%)	*N* = 321 (%)	*N* = 964 (%)	
Gender	Female	214 (64.5)	118 (35.5)	332 (41.0)	0.681
	Male	316 (66.1)	162 (33.9)	478 (59.0)	
Age category (in years)	<1	227 (66.4)	115 (33.6)	342 (40.1)	0.260
	1–3	169 (71.9)	66 (28.1)	235 (27.6)	
	3–6	130 (65.7)	68 (34.3)	198 (23.2)	
	>6	47 (61.0)	30 (39.0)	77 (9.04)	
Animal group	Pets	586 (65.1)	312 (34.9)	898 (93.6)	<0.001
	Livestock	55 (90.2)	6 (9.8)	61 (6.4)	
Clinical symptoms of rabies	No	94 (35.2)	173 (64.8)	267 (27.7)	<0.001
	Yes	534 (80.1)	133 (19.9)	667 (69.2)	
Biting animal	No	97 (53.0)	86 (47.0)	183 (19.7)	<0.001
	Yes	529 (70.7)	219 (29.3)	748 (80.3)	
History of rabies vaccination	No	598 (68.1)	280 (31.9)	878 (93.8)	<0.001
	Yes	22 (37.9)	36 (62.1)	58 (6.20)	
Circumstances of death	Euthanized	305 (69.3)	135 (30.7)	440 (53.0)	0.110
	Natural death	249 (63.8)	141 (36.2)	390 (47.0)	
Submitting person	Veterinarian	256 (74.9)	86 (25.1)	342 (35.5)	<0.001
	Other^*^	387 (62.2)	235 (37.8)	622 (64.5)	

^*^Usually the biting victim or owner of the animal.

a Direct fluorescent antigen test is the reference technique. For any negative test result, a second test is performed: either an isolation attempt in cell culture, or RT-PCR.

b Statistical significant difference calculated by chi square test.

## 4. Discussion

Over the 11 years of the study period, 66.7% of animal samples suitable for testing were positive for rabies. Overall, the positivity rate during this period increased in comparison to the previous report (48.9%; 220/450; 2005–2010) (12) and as compared to the period from 1959–1991 (57%;1416/2475) (p value *< 0.001*) (17). This high rate combined with the very limited number of districts submitting samples are indicative of underreporting and suggest that we are only measuring the “tip of the iceberg,” both for animal and human data. In livestock, only 53 samples were submitted over 11 years, however we suspect that the rabies incidence in cattle is higher and very few samples are submitted, as the percentage of positivity indicates a potentially high incidence of rabies in these species and transmission most likely occurs through dog bites. In fact, requests for diagnosis mainly come from persons who had been exposed to cattle bites and underwent post-exposure treatment or in the event of a cluster of suspect cases in a cattle herd due to biting behavior or deaths, which is often the case for domestic livestock. Clustered cases of rabies are unlikely to be identified as only one sample of a suspect case is usually sent to the NRL and reported by laboratory rabies surveillance, which may also explain the low number of livestock samples in this report.

In 2011–2012, the NRL received an increasing number of samples with a higher positivity rate. This pattern was repeated in 2016. However, without data on population size, it is not possible to calculate the incidence or confirm the epidemic cycle with a 3 to 6-year period of rabies circulation in Madagascar, as previously suggested by Hampson et al. (18) (Figure 2).

For dogs, the majority of diagnostic requests were made following a bite event. Most veterinarians or citizens do not keep a biting dog for observation as required by law but kill it immediately with or without taking samples or ignore it (submitted). Despite the regulations for the observation of the biting animals, low access to veterinarians, the lack of adequate infrastructure for this purpose and the high uncovered costs of this intervention lead the population to kill or ignore the biting animal.

It is noteworthy that animal owners were more inclined to send an animal sample to the NRL than veterinarians, indicating a lack of implication of veterinarians in the passive surveillance system. This may be explained by the fact that veterinarians are primarily focused on livestock in Madagascar (submitted) and are therefore more involved in sending suspect samples when livestock are involved. If they request a rabies diagnosis, their aim is to confirm rabies infection for their own information and for their clients in an agricultural context, rather than for surveillance and public health purposes. The challenges veterinarians face with the current surveillance system needs to be better understood and addressed to improve their role in rabies control.

Out of 987 samples, 23 could not be tested due to their state of conservation. Although this number is limited, it indicates either a lack of information on the correct handling of samples or a lack of means to send these samples correctly. The cost of transport to the NRL is covered by the veterinarian or the animal’s owner. This is certainly one of the main reasons for under-reporting. To avoid high transport costs, the NRL team has organized training courses on sampling techniques to limit the sending of large samples (brain sample vs. animal head). This led to a radical change in the type of samples sent to the NRL from 2021 onwards. However, even though all vets sent brain biopsies in 2021, surveillance coverage has not improved.

Information from rabies surveillance in Madagascar came mainly from the capital region where the NRL is located, and more than half of the districts remain “silent” about rabies. The lack of information on the occurrence/importance of rabies in these “data-less” areas leads to an erroneous perception of the absence of rabies in these regions.

The few control activities, such as dog culling and mass vaccinations conducted so far were in known “rabid” districts. Unfortunately, evaluation of these activities in terms of rabies incidence reduction is not available.

While Rajeev et al. (19, 20) estimated a human rabies incidence of 768 cases per year, the NRL only received an average of only four human samples per year (48 in total) over the 2011–2021 period. These samples were mainly sent by two medical services, indicating a lack of compliance with the surveillance system by other medical structures. However, when a case of rabies is suspected, the clinician’s assignment is limited to the management of the patient and public health reporting is therefore not a priority for them. In this context, it is vital to clarify roles and communication paths in the legal text and to make clinicians aware of the importance of confirming the diagnosis. In addition, one of the main causes of non-reporting could be the conflict over how to handle suspected human cases and their family (4). In the case of such a deadly disease, the family usually decides not to wait for the patient’s death in the hospital for financial and administrative reasons, as transporting a corpse is more difficult. Moreover, although the risk of human-to-human transmission is null, family members potentially exposed to bodily fluid and healthcare workers are concerned about contracting rabies during care. Their concerns must be addressed not only to ensure the best possible care for patients suspected of having rabies, but also to maintain a solid relationship between different people involved in surveillance (21). Clinicians need to be informed about how to collect, package and transport appropriate specimens, and on the importance of explaining to the patient’s family why specimen collection is necessary.

Eliminating rabies in dogs is the optimal control method for preventing the spread of the disease (2, 22). Actually, vaccination of dogs and control of stray dog populations are more efficient and cost effective than post-bite treatment in humans (2). However, most dogs in Madagascar are not vaccinated against rabies. While cultural factors (the dog is considered an unimportant or even “dirty” animal, not worthy of treatment) contribute to this situation, the fact that PEP is free certainly reduces the pressure to implement control measures in animals. Such phenomenon has also been observed in some communities in Chad (23). The “One Health” approach to rabies surveillance and control still needs to be implemented and awareness on this concept among stakeholders needs to be reinforced. It is still necessary to advocate among stakeholders for the absolute necessity of improving surveillance and control of rabies. Politicians have to understand the importance of funding to eliminate rabies in Madagascar. In 2023 a national strategic plan for rabies control was adapted and is a first step towards the goal of eliminating dog-transmitted human rabies by 2030.

In conclusion, rabies surveillance remains a challenge in Madagascar, mainly in terms of coverage and reporting. The activities carried out in response to a positive case of animal/human rabies focus on bitten victims or people potentially exposed to the rabid animal. There is a clear need to better understand the reasons for underreporting and to prioritize rabies surveillance, prevention and control in Madagascar, with improvements in budget, education and infrastructure. A more focused rabies control program, in the area of public health (information, awareness) and animal health, including vaccination of at least 70% of the dog population, is urgently needed to achieve the goal of eliminating dog-transmitted human rabies by 2030.

## Data availability statement

The data analyzed in this study is subject to the following licenses/restrictions: Dataset will be provided on demand from the corresponding author. Requests to access these datasets should be directed to soafy@pasteur.mg.

## Ethics statement

Ethical approval was not required for the study involving humans in accordance with the local legislation and institutional requirements. Written informed consent to participate in this study was not required from the participants or the participants' legal guardians/next of kin in accordance with the national legislation and the institutional requirements. Ethical approval was not required for the study involving animals in accordance with the local legislation and institutional requirements because we used anonymized laboratory surveillance data.

## Author contributions

SA: Conceptualization, Methodology, Supervision, Writing – original draft, Writing – review & editing, Formal analysis, Project administration, Resources, Visualization. MV: Conceptualization, Formal analysis, Methodology, Writing – review & editing. NiR: Supervision, Writing – review & editing. DR: Supervision, Writing – review & editing. MR: Investigation, Writing – review & editing. TR: Investigation, Writing – review & editing. LN: Investigation, Writing – review & editing. NaR: Supervision, Writing – review & editing. MA: Supervision, Writing – review & editing. HG: Conceptualization, Formal analysis, Methodology, Visualization, Writing – original draft, Writing – review & editing. VL: Conceptualization, Methodology, Supervision, Writing – original draft, Writing – review & editing. AD: Conceptualization, Methodology, Supervision, Writing – original draft, Writing – review & editing.
